# Investigation of acquired dysfibrinogenaemia in adult patients with sepsis using fibrinogen function vs. concentration ratios: a cross-sectional study

**DOI:** 10.3389/fmed.2023.1294301

**Published:** 2023-12-13

**Authors:** Rosa Toenges, Michael Steiner, Christian Friedrich Weber, Wolfgang Miesbach

**Affiliations:** ^1^Department of Medicine, Hemostaseology, University Hospital, Goethe University Frankfurt, Frankfurt, Germany; ^2^Medizinisches Labor Rostock, Rostock, Germany; ^3^Department for Anesthesiology, Intensive Care Medicine and Pain Therapy, University Hospital, Goethe University Frankfurt, Frankfurt, Germany; ^4^Department for Anesthesiology, Intensive Care Medicine and Emergency Medicine, Asklepios Clinics Hamburg, AK Wandsbek, Hamburg, Germany

**Keywords:** fibrinogen, dysfibrinogenaemia, sepsis, disseminated intravascular coagulation (DIC), clauss/RID fibrinogen ratios, prothrombin time-derived/RID ratios

## Abstract

**Introduction:**

Inherited or acquired molecular abnormalities form a clinically heterogeneous group of fibrinogen disorders called dysfibrinogenaemia. Apart from a pediatric case report and in contrast to other clinical conditions, acquired dysfibrinogenaemia has not been previously reported in septic patients.

**Methods:**

In an observational cohort study, 79 adult septic patients were investigated for the presence of acquired dysfibrinogenaemia at the time of their admission to the intensive care unit (ICU) of the University Hospital Frankfurt. Following established recommendations, fibrinogen clotting activity vs. antigen ratios were analyzed using Clauss fibrinogen, prothrombin-derived fibrinogen, and radial immunodiffusion (RID) fibrinogen concentration.

**Results:**

Prothrombin-derived fibrinogen levels were highest (527 ± 182 mg/dL) followed by Clauss fibrinogen (492 ± 209 mg/dL) and radial immunodiffusion fibrinogen (426 ± 159 mg/dL). Very few cases demonstrated hypofibrinogenaemia making overt disseminated intravascular coagulation (DIC) unlikely in the cohort investigated. Clauss/RID fibrinogen ratios were lower (1.17 ± 0.19) compared to prothrombin time-derived/RID ratios (1.35 ± 0.33). Using the Clauss/RID dataset, 21% of patients (16/76 patients) demonstrated values below a threshold ratio for suspected acquired dysfibrinogenaemia arbitrarily set at 1.0. In contrast, prothrombin-derived ratios were below the threshold in only 7% (4/58 patients).

**Discussion:**

The results point to the presence of acquired dysfibrinogenaemia in part of adult septic patients. If confirmed in further studies, this may form part of a specific laboratory signature of a sepsis-associated coagulation phenotype.

## 1 Introduction

Fibrinogen is a critically important plasma protein expressed by hepatocytes as a hexameric glycoprotein. Its main functions in primary and secondary hemostasis include fibrin clot formation, fibrin crosslinking by factor XIIIa, platelet aggregation, and fibrinolysis. Being a positive acute phase reactant, fibrinogen levels may increase 2–4-fold during an inflammation response.

Inherited or acquired structural abnormalities of fibrinogen are collectively referred to as dysfibrinogenaemia and may interfere with any of its multiple functions (1). Congenital fibrinogen disorders are rare and include hypo- and afibrinogenaemia, dysfibrinogenaemia, and combinations thereof. Clinical presentations are dominated by bleeding events of varying severity. However, thrombotic events occur in up to 20% of patients. The most common acquired fibrinogen disorder is hypofibrinogenaemia typically resulting from consumptive coagulopathy or hemodilution. Acute or chronic liver disease with reduced liver synthesis may also cause and contribute to hypo(dys)fibrinogenaemia. Additional causes of acquired dysfibrinogenaemia include drug therapy, malignancy, and the presence of anti-fibrinogen antibodies in autoimmune disorders. Patients may be asymptomatic or present with either bleeding and/or thrombotic events (2, 3).

The laboratory approach to congenital or acquired dysfibrinogenaemia relies on the use of different fibrinogen assays with the aim to demonstrate a critical difference between functional and antigen concentration levels. Screening tests including thrombin time and reptilase time may hint at the presence of dysfibrinogenaemia but are not performed routinely in the clinical laboratory. The diagnosis of dysfibrinogenaemia can be established by obtaining a fibrinogen activity vs. antigen ratio (4). For this purpose, most commonly thrombin-clottable fibrinogen is determined by the Clauss method or the fibrinogen activity is derived from the prothrombin time polymerization curve. Immunoreactive fibrinogen can be determined by different immunologic methods including enzyme-linked immunosorbent assays, turbidimetry/nephelometry, or radial immunodiffusion (5). Method-specific reference intervals should be considered and have an impact on resulting activity-antigen ratios. The diagnosis of inherited dysfibrinogenaemia can be verified at the molecular level using genetic analysis. In contrast, acquired dysfibrinogenaemia is considered a transient phenomenon with no specific molecular abnormalities (6).

Sepsis is a complex syndrome resulting from a dysregulated host response to infection. Increasing incidence and a high number of fatal outcomes are hallmarks of this life-threatening condition. Coagulation abnormalities have long been recognized to be present in virtually all patients with sepsis. They range from minimal activation of coagulation to overt disseminated intravascular coagulation (DIC) and variably contribute to sepsis morbidity and mortality. Diagnostic criteria for DIC were first established in 2001 and supplemented in 2019 with sepsis-induced coagulopathy (*SIC*) scoring system to detect the compensated phase of DIC. Hypofibrinogenaemia (<100 mg/dL) is an established diagnostic criterion for the overt DIC scoring system whereas no *SIC* range has as yet been added (7). Dysfibrinogenaemia is not included in the currently available scoring systems. Apart from a pediatric case report (8), the issue of acquired dysfibrinogenaemia in sepsis has not been approached. The aim of the present study was to investigate a clinical cohort of adult patients fulfilling the diagnostic criteria of sepsis for the presence of acquired dysfibrinogenaemia at the time of their admission to the intensive care unit (ICU) using three different fibrinogen assays and establishing fibrinogen activity-antigen ratios.

## 2 Materials and methods

### 2.1 Patients and study design

The present observational study was approved by the local ethics committee (protocol number 368/2010), and informed consent was obtained from all patients or their legal representatives, respectively. We included 79 adult patients, fulfilling the diagnostic criteria of sepsis according to the Consensus Criteria Sepsis-3 (9, 10), consecutively admitted to the ICU of the University Hospital Frankfurt. A cohort of 60 males and 19 females, aged between 18 and 80 years, was assessed for acquired dysfibrinogenaemia. Except for thromboprophylaxis with fixed-dosed subcutaneous low molecular weight heparin to 58 patients (74%), no further coagulation-directed drug therapy was administered. Patient characteristics are summarized in Table 1.

**Table 1:** Characteristics of a cohort of 79 septic patients, at the time of admission to the intensive care unit.

Patient characteristics	Total cohort (*n* = 79)
Age, median, years	62
Sex, male, %	67
Reason for admission	
Cardiac	16
Hepatic	5
Abdominal	18
Renal	2
Pulmonary	30
Hematologic	3
Oncologic	5
Comorbidities, median (range)	3 (1–5)
Comorbidity conditions, % (*n*)	
Diabetes mellitus	22 (17)
Chronic pulmonary disease	11 (9)
Chronic kidney disease	19 (15)
Coronary heart disease	43 (34)
Malignancy	13 (10)
Liver disease	15 (12)
Neurologic disease	11 (9)
Rheumatoid arthritis/autoimmune disorder	6 (5)
Thromboprophylaxis, % (*n*)	74 (58)

### 2.2 Fibrinogen assays

Plasma samples were obtained at the time of admission to the ICU from venous citrate-anticoagulated blood and stored in aliquots at −80°C. Hemolytic, lipaemic, icteric, and turbid plasma samples were excluded for their potential interference with the fibrinogen assays. Samples demonstrating hemolysis, Thrombin-clottable fibrinogen was assessed using Clauss method reagents (STA Fibrinogen). Prothrombin time (PT)-derived fibrinogen was obtained from PT curves using PT reagents (STA Neoplastin Plus). The coagulation analyzer STAR-R Evolution (Stago) was used in automated mode according to the manufacturer’s instructions including internal and external quality control assessment. Immunoreactive fibrinogen was analyzed by radial immunodiffusion using NOR-Partigen fibrinogen plates (Siemens), and readings were obtained at 18 and at 48 h after incubation, respectively, to not miss late diffusion endpoints. Fibrinogen ratios were calculated using Clauss fibrinogen and PTder fibrinogen, respectively, divided by RID fibrinogen. The “relative fibrinogen deficit” was obtained by subtracting fibrinogen activity from fibrinogen concentration.

## 3 Results

Using three methods, fibrinogen data were obtained from 79 adult septic patients consecutively admitted to the ICU. Two assays, clottable fibrinogen using the Clauss assay and prothrombin time-derived (PTder) fibrinogen, reflect functional fibrinogen levels whereas fibrinogen antigen concentration was determined by radial immunodiffusion (RID). For the lack of sufficient sample volume, prothrombin time-derived fibrinogen could not be analyzed for 21 patients. Prothrombin time-derived fibrinogen levels were highest (527 ± 182 mg/dL) followed by Clauss fibrinogen (492 ± 209 mg/dL), and radial immunodiffusion (426 ± 159 mg/dL). Hypofibrinogenaemia defined as fibrinogen below 100 mg/dL was detected in two cases by Clauss and RID assays, respectively. PTder fibrinogen was not below 100 mg/mL. Conversely, the proportion of hyperfibrinogenaemia defined as fibrinogen above 450 mg/dL was highest using PTder fibrinogen (65.5%) followed by Clauss fibrinogen (54.5%) and RID fibrinogen (48.7%; Table 2).

**Table 2:** Fibrinogen data obtained from adult septic patients (*n* = 79).

	Clauss	PTder	RID
*n*	77	58	78
Mean ± SD (mg/dL)	492 ± 209	527 ± 182	426 ± 159
Hypofibrinogenaemia (< 100 mg/dL), *n* (%)	2 (2.6)	n.d.	2 (2.6)
Range (mg/dL)	66–70		50–93
Hyperfibrinogenaemia (> 450 mg/dL), *n* (%)	42 (54.5)	38 (65.5)	38 (48.7)
Range (mg/dL)	457–1,026	453–940	452–736

Fibrinogen was analyzed as clottable fibrinogen (Clauss), as prothrombin time-derived fibrinogen (PTder), and as immunoreactive fibrinogen using radial immunodiffusion (RID). n.d., none detected.

**Figure 1:**
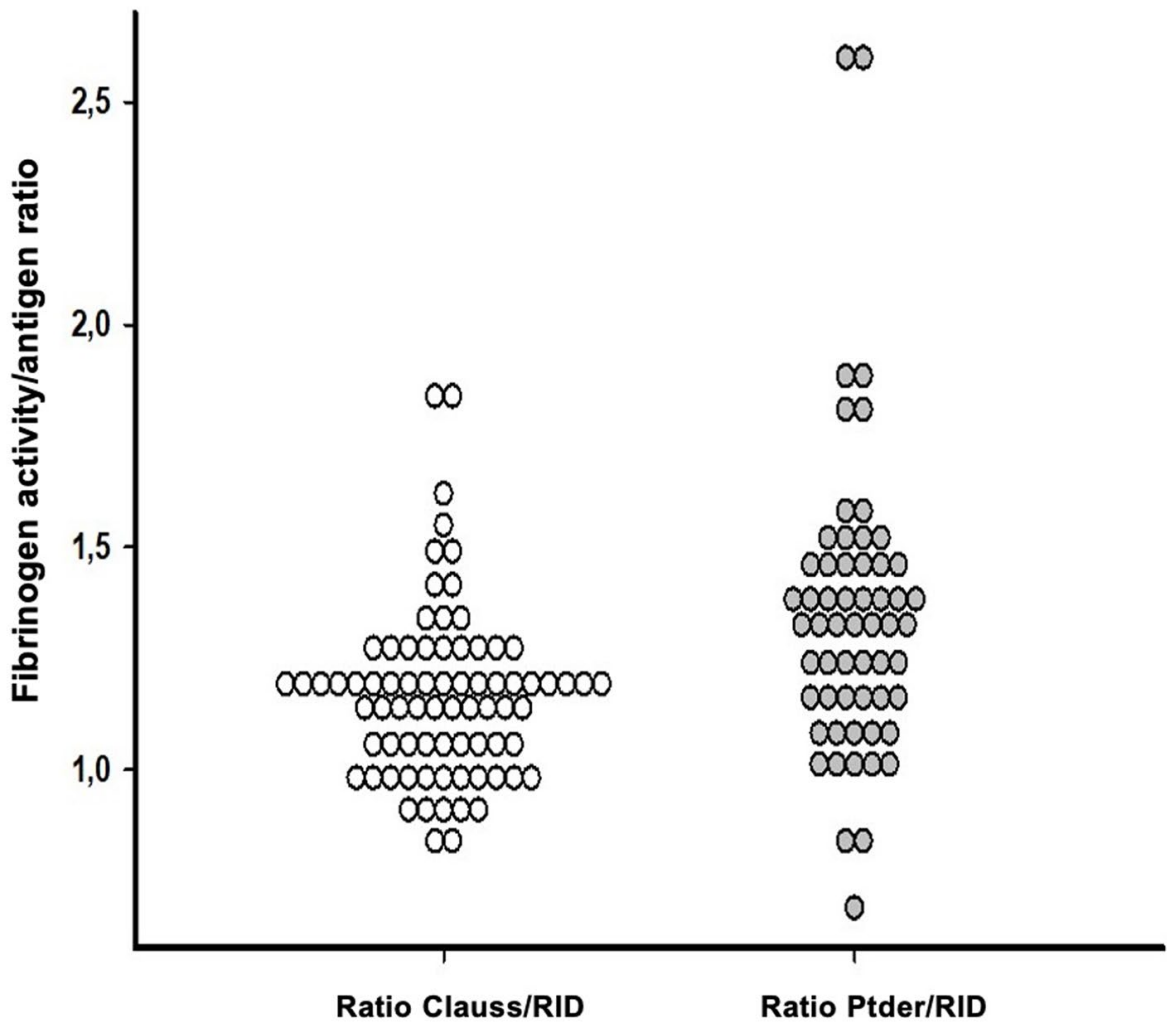
Fibrinogen activity vs. antigen ratios were calculated using Clauss fibrinogen (white) or PTder fibrinogen (gray), divided by RID in adult septic patients. Clauss/RID fibrinogen ratios were lower (1.17 ± 0.19, *n* = 76) compared to PTder/RID fibrinogen ratios (gray; 1.35 ± 0.33, *n* = 58). Ratios below the threshold of 1 were suspected as being a possible indicator for dysfibrinogenemia. PTder, Prothrombin time-derived fibrinogen; RID, Radial immunodiffusion (RID).

We next calculated fibrinogen activity vs. antigen ratios using Clauss fibrinogen and PTder fibrinogen, respectively, divided by RID fibrinogen. Clauss/RID fibrinogen ratios were lower (1.17 ± 0.19, *n* = 76) compared to PTder/RID fibrinogen ratios (1.35 ± 0.33, *n* = 58). Giving the expectation that fibrinogen activity should align with its concentration, a threshold ratio for suspected dysfibrinogenaemia was arbitrarily set at 1.0 with lower ratios being a possible indicator for the presence of dysfibrinogenemia. Using the Clauss/RID fibrinogen dataset, 16 of 76 ratios (21%) were below the threshold ranging from 0.81 to 0.99. In contrast, the PTder/RID fibrinogen dataset yielded only 4 of 58 ratios (7%) below the threshold ranging from 0.69 to 0.99 (Figure 1). The corresponding “relative fibrinogen deficit” (fibrinogen concentration minus fibrinogen activity) was between 4 and 158 mg/dL.

## 4 Discussion

The present observational cohort study aimed to investigate the presence of acquired dysfibrinogenaemia in adult septic patients at the time of their admission to the ICU. Fibrinogen clotting activity vs. antigen ratios were calculated for 97 patients. Using the Clauss fibrinogen/radial immunodiffusion dataset, 21% of patients (16/76 patients) demonstrated values below a threshold ratio for suspected acquired dysfibrinogenaemia. Prothrombin-derived fibrinogen ratios were below the threshold in only 7% (4/58 patients). Collectively, these results point to the occurrence of acquired dysfibrinogenaemia in part of adult septic patients.

Dysfibrinogenaemia comprise a group of inherited or acquired structurally abnormal fibrinogen molecules with variable pathophysiology and clinical features (1). Knowledge on inherited dysfibrinogenaemia has been steadily accumulating with most details gained from studies of rare bleeding disorders (3). In contrast, the issue of acquired dysfibrinogenaemia has not been comprehensively addressed in the past. Most studies were performed in patients with liver or biliary tract disease, with fewer studies and case reports on its presence in malignancy and other clinical conditions. The pathophysiology of acquired dysfibrinogenaemia in liver disease involves increased sialylation of carbohydrate side chains whereas cancer-associated dysfibrinogen is supposed to be synthesized and secreted by tumor cells (7). The pathogenesis of acquired dysfibrinogenaemia in sepsis has not been formally approached in studies. We propose that it may involve inflammation-induced abnormal glycosylation of plasma fibrinogen and/or yet unidentified additional posttranslational protein modifications. Clinically, it remains largely unknown if acquired dysfibrinogenaemia contributes to either bleeding or thrombotic events in addition to the disease-specific bleeding or thrombotic risk.

Being an example of significant cross talk between inflammation and coagulation, sepsis-induced coagulopathy is a continuum ranging from subclinical coagulopathy to overt, potentially fatal disseminated intravascular coagulation (DIC) (11) with a severely deranged hemostatic balance. Diagnostic criteria for overt, decompensated DIC were first established in 2001 and recently supplemented by the sepsis-induced coagulopathy (*SIC*) scoring system to allow for detecting earlier stages of compensated DIC in sepsis (7). With regard to fibrinogen, hypofibrinogenaemia defined as values below 100 mg/dL is only included in the overt DIC diagnostic scoring system. To the authors’ best knowledge, the presence of acquired dysfibrinogenaemia in sepsis has not been systematically studied in the past. Only one study investigated a fibrinogen function vs. antigen ratio in sepsis and concluded that ratios were largely aberrant in septic patients compared to normal values with plasmin-mediated fibrin(ogen) breakdown products possibly contributing to over-proportionally decreased fibrinogen function versus antigen ratios (12).

We conducted an observational cohort study in adult septic patients at the time of their admission to the intensive care unit. Fibrinogen was investigated using three different methods. Hypofibrinogenaemia defined as values below 100 mg/dL were observed in only very few patients. As this finding would be consistent with overt DIC, this advanced sepsis-induced hemostatic derangement could be excluded for the majority of the patients investigated. However, it should be noted that fibrinogen was assessed only at a single time point instead of being followed sequentially (13).

Using a generally accepted diagnostic approach to suspected dysfibrinogenaemia employing a fibrinogen clotting activity to antigen ratio (4), we investigated its presence in 79 sepsis patients at the time of their admission to the intensive care unit. Fibrinogen assays were selected according to their availability in a routine coagulation laboratory. It should be emphasized that several methods have been developed to determine fibrinogen in plasma (5). Assays vary in technical principles, equipment and reagents requirements, degree of expertise, and turnaround time, to name but a few. Under routine conditions, most laboratories use either the Clauss fibrinogen assay or a prothrombin time-derived fibrinogen estimation. The Clauss assay’s readout is clot formation (14) and it is generally accepted as a measure of clottable fibrinogen. Fibrinogen estimation derived from the prothrombin time is an attractive alternative as it provides a fibrinogen value at no extra time and cost (15). Depending on the method used and the cohort of patients under consideration, PT-derived fibrinogen concentrations have been reported increased compared to the Clauss method with varying discrepancies (6). Correlations between either fibrinogen value or *in vivo* hemostasis remain difficult to judge. True clottable fibrinogen assays are time consuming and technically difficult precluding their routine use. A number of immunological assays are available to measure fibrinogen concentration including ELISA, radial immunodiffusion, and nephelometry. The disadvantage for clinical interpretation is that a protein concentration is measured rather than functional activity. However, it is exactly this combination of measures that is required to investigate inherited or acquired dysfibrinogenaemia (4). Harmonization efforts notwithstanding (16), no consensus has been reached as to which methods should be preferentially employed for the investigation of dysfibrinogenaemia (17).

Confirming data from previous investigations, the present study yielded discrepant fibrinogen values being highest for PT-derived fibrinogen followed by Clauss fibrinogen and radial immunodiffusion. Accordingly, the percentage of hyperfibrinogenaemia ranked in the same order. Increased fibrinogen in a substantial number of septic patients is expected to result from its behavior as a positive acute phase reactant.

The focus of the present study was to investigate the presence of acquired dysfibrinogenaemia in adult septic patients using the concept of calculated fibrinogen activity versus antigen ratios. Clauss/RID ratios were lower compared to PT-derived/RID ratios. Applying a threshold ratio of 1.0 for suspected dysfibrinogenaemia, the Clauss/RID approach yielded 21% values below threshold compared to 7% using the PT-derived/RID ratio (Figure 1). It may be argued that the acute phase response would have a critical impact on the ratio concept. However, it has been previously demonstrated that the fibrinogen function vs. antigen concentration ratio can be applied regardless of the presence of an acute phase reaction (18). In contrast to previous studies using a fibrinogen activity vs. antigen ratio threshold of 0.7 to identify dysfibrinogenaemia, the present analysis considered a threshold ratio of 1.0 with the aim to demonstrate substantial ratio heterogeneity within the dataset (Figure 1), and identified a subset of patients with evidence of dysfibrinogenaemia. Receiver operating characteristic (ROC) analyses to compare different threshold ratios were beyond the scope of the present analysis.

If validated by independent studies, our results point to a significant proportion of adult septic patients displaying evidence of acquired dysfibrinogenaemia. It should be emphasized that this potential hemostatic derangement occurs before fibrinogen consumption reaches the limit of hypofibrinogenaemia as defined by values below 100 mg/dL and may therefore be part of sepsis-induced coagulopathy (*SIC*). Data from the present study apply only for the methods employed as variability among fibrinogen methods is well known (5) and have an impact on calculated ratios between any two of them. This is further exemplified in the present study by the varying proportion of acquired dysfibrinogenaemia depending on the fibrinogen activity assay used for calculating ratios. Future studies may consider cross-calibrating various fibrinogen assays used. This would equally apply to both functional as well as immunological assays. However, this approach would be beyond the manufacturers’ assay specification and require validation efforts. Nevertheless, improved comparability between fibrinogen assays may thus be obtained and equimolarity with a fibrinogen activity vs. antigen ratio of 1.0 may form the basis for future studies in both inherited and acquired dysfibrinogenaemia. A different approach has recently been selected with a fibrinogen ratio obtained from two functional assays to identify inherited dysfibrinogenaemia (19) However, this concept has not yet been employed for acquired dysfibrinogenaemia.

The functional implications of sepsis-induced acquired dysfibrinogenaemia remain unknown and call for confirmatory studies with a focus on fibrin structure and clot properties in septic patients. Viscoelastic tests including thromboelastography (TEG) and rotational thromboelastography (ROTEM) should be added in future *SIC* studies focusing on acquired dysfibrinogenaemia (20). We propose that acquired dysfibrinogenaemia in part of septic patients may belong to a laboratory signature of a sepsis-associated coagulation phenotype. It remains to be investigated if this will translate into a risk indicator for clinical outcome.

In contrast to inherited dysfibrinogenaemia, acquired dysfibrinogenaemia has been less extensively studied (1). Except for a pediatric case report (19), the lack of studies in sepsis appears surprising given the central role of fibrinogen in inflammation (21). Although not focusing on dysfibrinogenaemia, a recent study compared Clauss and PT-derived fibrinogen with specific categories of disease and concluded that both methods are not interchangeable for certain clinical conditions raising the possibility of dysfibrinogenaemia (22). Another study investigated hemostatic impairment in postpartum hemorrhage being a specific form of DIC and reported evidence of acquired (hypo)dysfibrogenaemia in acute obstetric coagulopathy (23). Collectively, these data call for further studies to more fully characterize the occurrence and clinical significance of acquired dysfibrinogenaemia in a wider variety of clinical settings.

Limitations of the present study include:

First, the study included only a single point of analysis at the time of admission of adult septic patients to the ICU. Dynamic studies with multiple analyses are required to follow the natural history of acquired dysfibrinogenaemia and specifically to answer the question if this condition will resolve with clinical improvement. Second, data reported in the present study apply only to the fibrinogen methods used and future studies should include additional fibrinogen methods for comparison. Specifically, more sophisticated immunological assays should be used to allow for high throughput and short turnaround times. Third, the possible importance of acquired dysfibrinogenaemia on *in vivo* hemostasis in septic patients is at present unknown and should be investigated in future studies by adding measures of fibrin and clot properties including thrombin and reptilase times, D-dimer, and/or other fibrin(ogen) degradation products. Similarly, clinical outcome measures should be determined in adult septic patients in the presence of acquired dysfibrinogenaemia.

## Data availability statement

The raw data supporting the conclusions of this article will be made available by the authors, without undue reservation.

## Ethics statement

The studies involving humans were approved by Institutional Review Boards of the UCT and the Ethical Committee at the University Hospital Frankfurt. The studies were conducted in accordance with or their legal representatives local legislation and institutional requirements. The participants provided their written informed consent to participate in this study.

## Author contributions

MS: Data curation, Formal analysis, Software, Writing – original draft. CW: Investigation, Project administration, Supervision, Writing – review & editing. WM: Conceptualization, Investigation, Methodology, Project administration, Resources, Supervision, Writing – review & editing. RT: Data curation, Formal analysis, Investigation, Methodology, Validation, Visualization, Writing – original draft, Writing – review & editing.
